# Differential efficacy of tyrosine kinase inhibitors according to the types of EGFR mutations and agents in non-small cell lung cancer: a real-world study

**DOI:** 10.1186/s12885-023-11782-6

**Published:** 2024-01-12

**Authors:** Tae-Hwan Kim, Jin-Hyuk Choi, Mi Sun Ahn, Hyun Woo Lee, Seok Yun Kang, Yong Won Choi, Young Wha Koh, Seung-Soo Sheen

**Affiliations:** 1 Departments of Hematology-Oncology, Ajou University School of Medicine, 164 Worldcup-ro, Yeongtong-gu, Suwon, 16499 Gyeonggi-do Korea; 2 Departments of Pathology, Ajou University School of Medicine, 164 World cup-ro, Yeongtong-gu, Suwon, Gyeonggi-do Korea; 3 Departments of Pulmonary and Critical Care Medicine, Ajou University School of Medicine, 164 World cup-ro, Yeongtong-gu, Suwon, Gyeonggi-do Korea

**Keywords:** Non-small cell lung cancer, Tyrosine kinase inhibitors, EGFR, Exon 19 deletion

## Abstract

**Background:**

Both first and second-generation EGFR-TKIs are recommended in advanced NSCLC with common EGFR mutations. However, there are few data on the difference in efficacy of EGFR-TKIs based on the type of EGFR mutation and agents.

**Methods:**

This retrospective real-world study evaluated the outcomes and clinicopathologic characteristics, including the type of EGFR mutations, of 237 advanced NSCLC patients treated with first- or second-generation (afatinib) EGFR-TKIs as first-line therapy.

**Results:**

The median progression-free survival (PFS) and overall survival (OS) of all patients were 11 months (M) and 25M, respectively. In the univariate analysis, patients with exon 19 deletion (del) (*n*=130) had significantly longer median OS compared to those with other mutations (L858R: 84, others: 23) (30 vs. 22 M, *p*=0.047), without a difference in PFS (*p*=0.138). Patients treated with afatinib (*n*=60) showed significantly longer median OS compared to those treated with first-generation TKIs (gefitinib: 159, erlotinib: 18) (30 vs. 23 M, *p*=0.037), without a difference in PFS (*p*=0.179). In patients with exon 19 del, there was no significant difference in median PFS (*p*=0.868) or OS (*p*=0.361) between patients treated with afatinib and those treated with first-generation TKIs, while significantly better PFS (*p*=0.042) and trend in OS (*p*=0.069) were observed in patients receiving afatinib in other mutations. Exon 19 del was independently associated with favorable OS (*p*=0.028), while age >70 years (*p*=0.017), ECOG performance status ≥2 (*p*=0.001), primary metastatic disease (*p*=0.007), and synchronous brain metastasis (*p*=0.026) were independent prognostic factors of poor OS.

**Conclusions:**

The EGFR exon 19 del was associated with favorable OS in advanced NSCLC patients receiving first-line EGFR-TKIs. Moreover, in patients with exon 19 del, first-generation TKIs seem to be a reasonable treatment option if osimertinib is unavailable.

## Background

Non-small cell lung cancer (NSCLC) is one of the most common neoplasms, and the leading cause of cancer-related mortality in several countries, including South Korea [1–3]. Approximately 65% of patients with NSCLC are diagnosed with advanced status [3], and the clinical outcome of advanced NSCLC with a median overall survival (OS) of 11–22 months remains unsatisfactory, despite advances of palliative chemotherapy [2, 4].

Since NSCLC patients with epidermal growth factor receptor (EGFR)-activating mutations (exon 19 deletion and exon 21 L858R mutation), which are observed in 20–40% of Asian patients, demonstrated high response to EGFR tyrosine kinase inhibitors (TKIs) [5, 6], EGFR-TKIs have been considered as the standard first-line therapy of EGFR mutation-positive advanced NSCLC [2, 7]. In the NCCN [8] and ESMO guidelines [9], osimertinib, third-generation TKI, is recommended as a first-line treatment based on the results of FLAURA trial, which reported a significant prolongation of progression-free survival (PFS) compared to first-generation EGFR-TKIs (gefitinib, erlotinib) [10]. However, the OS benefit of osimertinib was rather marginal (median OS: 38.6 vs. 31.8 months, *P* = 0.046), and there was no OS benefit for Asian patients and those with EGFR L858R mutation [11]. Therefore, first and second-generation EGFR-TKIs are still recommended equally, especially for Asian patients. Although there are some real-world data on the comparison of outcomes between first- and second-generation EGFR-TKIs [12–16], only two trials have compared the efficacy of first-line first- and second-generation EGFR-TKIs, reporting conflicting results [17–20].

The presence of differences in sensitivity to EGFR-TKIs among various types of EGFR mutation remains a subject of debate. Several studies demonstrated longer PFS and/or OS in patients with exon 19 deletion compared to those with L858R mutation [15, 21–29]. On the other hand, no significantly different effect of EGFR-TKIs based on the types of EGFR mutation **was** observed in other retrospective studies and phase III trials [30–36]. In addition, many studies included patients who received EGFR-TKIs as variable lines [22, 23, 25, 29, 30].

Therefore, in the present study, the clinical outcomes of EGFR mutation-positive advanced NSCLC patients treated with first- and second-generation EGFR-TKIs as their first-line treatment were investigated in terms of the EGFR mutation subtypes as well as the agents.

## Methods

### Study population

All EGFR mutation-positive advanced NSCLC patients who started first-line first- or second-generation EGFR-TKIs therapy between July 2011 and June 2018 at our institution were retrospectively identified. The eligibility criteria were cytologically or histologically confirmed NSCLC, and either stage IV based on the 7th edition of the American Joint Committee on Cancer (AJCC) [37] or stage IIIB/recurrent disease unsuitable for definitive local treatment. Some patients and methods of this study cohort were included in previous retrospective studies on EGFR-TKIs in NSCLC [21, 38]. Nonetheless the criteria for eligibility criteria of this study were slightly different from those of the previous studies, with longer follow-up duration of patients.

### Clinical review

The clinical information of eligible patients was retrospectively reviewed. Data collected on the patients included patient characteristics (age, gender, smoking history), performance status (PS) based on the Eastern Cooperative Oncology Group (ECOG) performance scale, histology, disease status at the start of EGFR-TKIs, presence of synchronous brain metastasis, second- or further-line of therapy, and information of survival status.

### EGFR mutation analysis

A direct sequencing method was applied for detecting EGFR mutation without routine tumor enrichment. Retrieved Formalin-fixed, paraffin embedded (FFPE) tumor samples were used for genomic DNA extraction by the QIAmp DNA FFPE Tissue Kit (Qiagen, Hilden, Germany). Polymerase chain reaction (PCR) amplification of EGFR exons 18 to 21, using intron-based primers was followed. Sequencing was performed in both the forward and reverse directions. Since September 2014, the peptide nucleic acid-locked nucleic acid (PNA-LNA) PCR clamp method has been applied in almost all cases. Genomic DNA of EGFR mutation hot-spots were amplified by PCR with a PNA clamp primer synthesized from a PNA with a wild-type sequence and detected by a fluorescent primer that incorporates locked nucleic acids. This method for preferential amplification of the mutant sequence can detect EGFR mutation in specimens containing 100 to 1000 excess copies of wild-type EGFR sequence [39].

### Statistical analysis

The Kaplan–Meier method was used for the calculation of OS and PFS. The time from the start day of the EGFR-TKI treatment to death and the time to disease progression or death by any cause were defined as OS and PFS, respectively. In case of surviving patients at the time of data cut-off with uncertain disease status, the data were censored on the last evaluation date at our institution for PFS. Data on the survivors were censored at the last follow-up for OS. The log-rank test **was** used for the analysis of the differences between the survival curves. Fisher’s exact test was applied to compare categorical variables among the different groups. The joint effects of several variables on survival were determined by the Cox proportional-hazards regression model, including factors with p-values < 0.1 in the univariate analysis. All statistical analyses were performed two-sided using SPSS for Windows 20.0 software.

### Statement of ethics

This research protocol was approved by the Institutional Review Board (IRB) of Ajou University Hospital, Suwon, Republic of Korea (AJOUIRB-MDB-2019-394) and all methods were performed in accordance with the relevant guidelines and regulations. The study was designed retrospectively. Written informed consent from patients was not required in accordance with guidelines of the IRB of Ajou University Hospital.

## Results

### Patient characteristics

A total of 237 EGFR-mutation-positive, advanced NSCLC patients, who received first- (gefitinib, erlotinib) or second-generation (afatinib) EGFR-TKIs as first-line palliative chemotherapy, were analyzed. Table 1 describes the clinicopathological characteristics of patients. Almost all patients underwent EGFR-TKI treatment in the routine practice, except for four patients who received gefitinib in a clinical trial as a first-line TKI. The median age of all patients was 67 (23–91), and 138 (58.2%) patients were female. Primary metastatic and recurrent disease were diagnosed in 199 (84%) and 38 (16%) patients, respectively. The ECOG PS was 0 or 1 in 194 (81.9%) patients, 2 in 31 patients, 3 in 11 patients, and 4 in 1 patient. Synchronous brain metastasis was identified in 77 (32.5%) patients. Among the 220 patients with disease progression after first-line EGFR-TKI treatment, 37 (16.8%) patients received third-generation EGFR-TKIs (osimertinib: 28, olmutinib: 9 patients) as second- (27 patients) or further-lines (10 patients).

**Table 1:** Patient characteristics

**Clinical characteristics**	**Exon 19 deletion (*n*=130)**	**Others (*n*=107)**	p*	**1st generation TKI (*n*=177)**	**Afatinib** **(*n*=60)**	p*
Age, years						
≤70	86 (66.2%)	65 (60.7%)	0.417	101 (57.1%)	50 (83.3%)	<0.0001
>70	44 (33.8%)	42 (39.3%)		76 (42.9%)	10 (16.7%)	
Gender						
Female	75 (57.7%)	63 (58.9%)	0.895	115 (65.0%)	23 (38.3%)	<0.0001
Male	55 (42.3%)	44 (41.1%)		62 (35.0%)	37 (61.7%)	
Smoking						
No	80 (61.5%)	59 (55.1%)	0.355	115 (65.0%)	24 (40.0%)	0.001
Yes	50 (38.5%)	48 (44.9%)		62 (35.0%)	36 (60.0%)	
ECOG PS						
0/1	108 (83.1%)	86 (80.4%)	0.615	139 (78.5%)	55 (91.7%)	0.021
≥2	22 (16.9%)	21 (19.6%)		38 (21.5%)	5 (8.3%)	
Brain metastasis						
No	88 (67.7%)	72 (67.3%)	1.000	120 (67.8%)	40 (66.7%)	0.874
Yes	42 (32.3%)	35 (32.7%)		57 (32.2%)	20 (33.3%)	
Disease status						
Recurrent	20 (15.4%)	18 (16.8%)	0.859	27 (15.3%)	11 (18.3%)	0.549
Primary metastatic	110 (84.6%)	89 (83.2%)		150 (84.7%)	49 (81.7%)	
Type of EGFR mutation						
Exon 19 deletion	-	-	-	88 (49.7)	42 (70.0)	0.007
Others	-	-		89 (50.3)	18 (30.0)	
3rd generation TKI after PD^a^						
Yes	31 (25.0%)	6 (6.2%)	<0.0001	23 (13.9%)	14 (25.9%)	0.058
No	93 (75.0%)	90 (93.8%)		143 (86.1%)	40 (74.1%)	

*ECOG* Eastern Cooperative Oncology Group, *PS* Performance status, *TKI* Tyrosine kinase inhibitor, *PD* Progressive disease, *M* Months (median), *p p*-value

*Fisher’s exact test

^a^Excluding 17 patients without documentation of PD

Direct sequencing (82 patients), the PNA-LNA PCR clamp method (152 patients), and next-generation sequencing (3 patients) were used for detection of EGFR mutation subtypes. The most common type of EGFR mutation was the exon 19 deletion (130 patients, 54.9%), followed by L858R mutation in exon 21 (84 patients, 35.4%). Moreover, 18 patients had uncommon mutations (exon 18 mutation: 8, exon 18 with exon 20 mutation: 3, exon 20 mutation: 3, exon 21 mutation: 4 [L861Q: 3, other mutation: 1]), and five patients dual mutations (exon 19 deletion with L858R mutation: 1, L858R mutation with exon 18 mutation: 1, exon 19 deletion with exon 20 mutation: 1, and L858R and L861Q mutations: 2). The baseline characteristics were not statistically different based on the EGFR mutation subtype. However, the proportion of patients who received third-generation TKIs after progression was significantly higher in patients with exon 19 deletion compared to those with other mutations (L858R and uncommon or dual mutations) (Table 1).

A total of 159 (67.1%), 18 (7.6%), and 60 (25.3%) patients were treated with gefitinib, erlotinib, and afatinib, respectively. The baseline characteristics of patients treated with afatinib in this cohort were significantly associated with younger age, male, smoker, better performance status, and exon 19 deletion (Table 1).

### Progression-free and overall survival

**Fig. 1:**
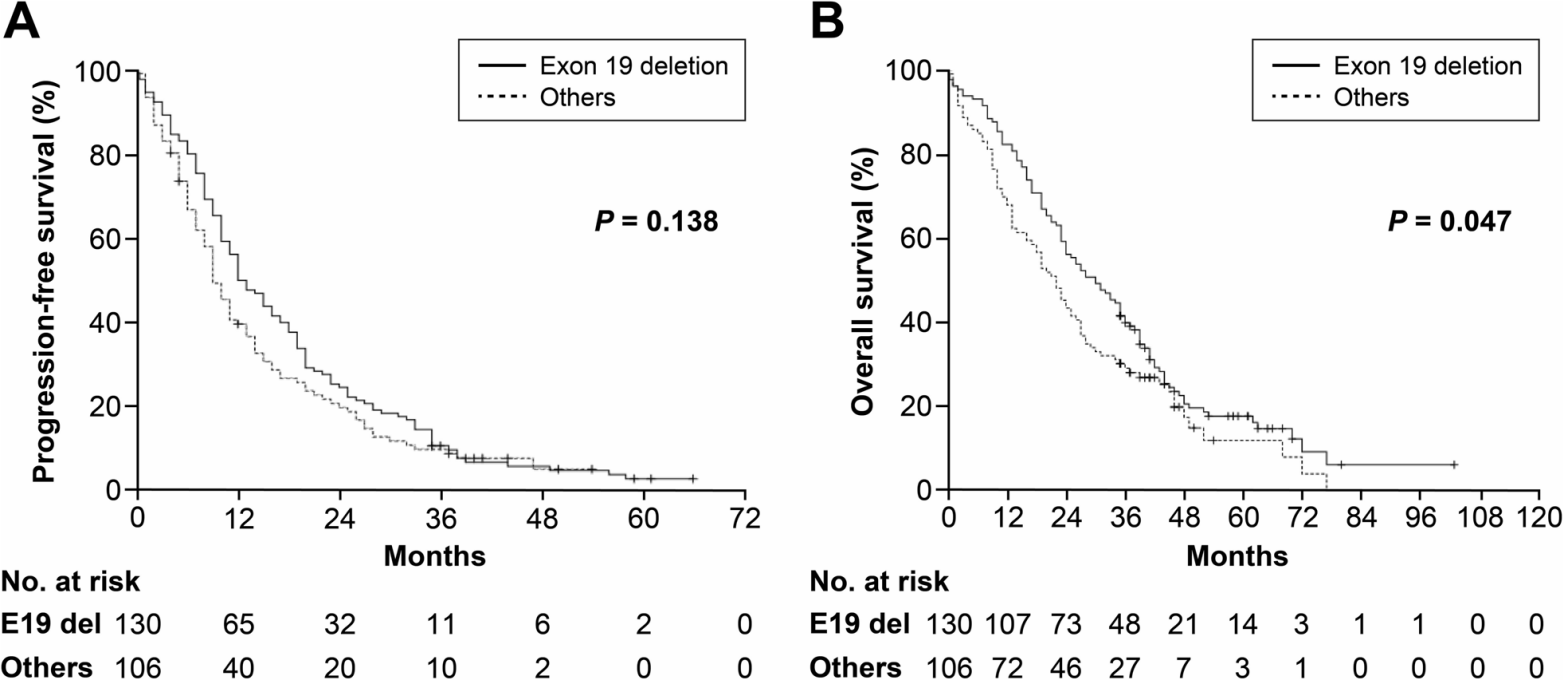
**A** Progression-free survival and (**B**) overall survival from the start of EGFR-TKI according to EGFR mutation subtypes. Exon 19 deletion: 130 patients, others: 106 patients (L858R: 83, uncommon mutations: 18, dual mutations: 5). Censoring was indicated by vertical lines

**Fig. 2:**
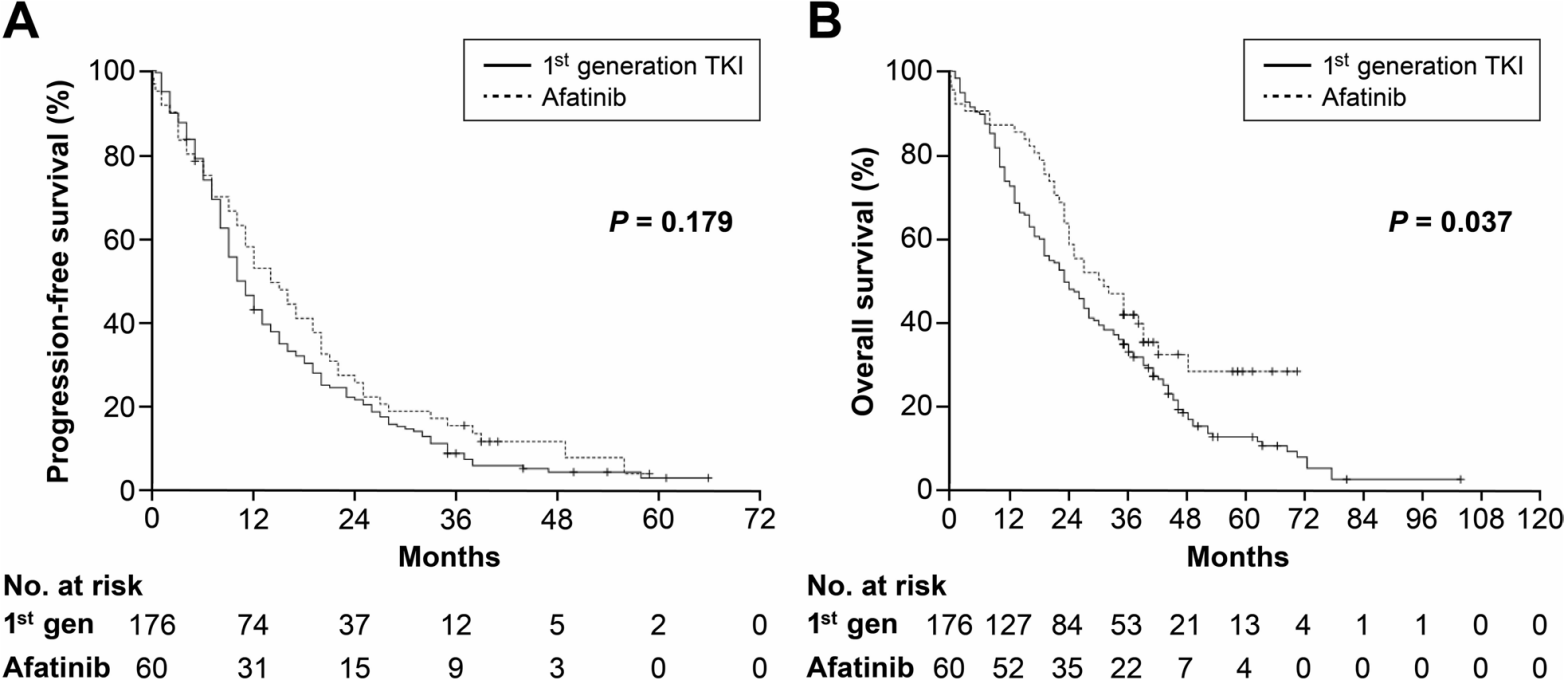
**A** Progression-free survival and (**B**) overall survival from the start of EGFR-TKI according to types of tyrosine kinase inhibitors. Exon 19 deletion: 130 patients, others: 106 patients (L858R: 83, uncommon mutations: 18, dual mutations: 5). Censoring was indicated by vertical lines

The median follow-up duration was 43 (35–103) months for the survivors (42 patients) at the time of analysis. Only one patient was lost to follow-up for survival status after receiving a 14-day prescription of gefitinib and was excluded from the analysis for PFS and OS. The median PFS and OS from the start of EGFR-TKI treatment for all patients were 11 and 25 months, respectively, while those for the 214 patients with EGFR-activating mutation were 12 and 26 months. Patients with exon 19 deletion had significantly longer median OS compared to those with other mutations (30 vs. 22 months, *p*=0.047, Fig. 1B), without a difference in PFS (12 vs. 9 months, *p*=0.138, Fig. 1A). Patients treated with afatinib showed significantly longer median OS (30 vs. 23 months, *p*=0.037, Fig. 2B) compared to those treated with first-generation TKIs, without a difference in PFS (14 vs. 10 months, *p*=0.179, Fig. 2A). In the multivariate analysis, EGFR exon 19 deletion showed independent association with favorable OS (*p*=0.028), while age >70 years (*p*=0.017), ECOG performance status ≥2 (*p*=0.001), primary metastatic disease (*p*=0.007), and synchronous brain metastasis (*p*=0.026) were independent prognostic factors for unfavorable OS (Table 2).

**Table 2:** Univariate and multivariate analysis of progression-free and overall survival

	**PFS**					**OS**				
**Clinical characteristics**	**M**	p*	**HR**	**95%CI**	***p*†**	**M**	p*	**HR**	**95% CI**	***p*†**
Age, years										
≤70	10.0	0.270				28.0	0.004	1		0.017
>70	14.0					22.0		1.47	1.07-2.01	
Gender										
Female	12.0	0.481				24.0	0.752			
Male	11.0					25.0				
Smoking										
No	12.0	0.365				27.0	0.359			
Yes	11.0					23.0				
ECOG PS										
0/1	13.0	<0.0001	1		0.001	29.0	<0.0001	1		0.001
≥2	7.0		1.80	1.26-2.56		12.0		1.88	1.28-2.75	
Brain metastasis										
No	14.0	<0.0001	1		0.001	30.0	<0.0001	1		0.026
Yes	9.0		1.65	1.22-2.23		19.0		1.46	1.05-2.02	
Disease status										
Recurrent	17.0	0.005	1		0.059	48.0	<0.0001	1		0.007
Primary metastatic	11.0		1.46	0.99-2.15		23.0		1.86	1.18-2.92	
Type of TKI										
1^st^ generation	10.0	0.179				23.0	0.037	1		0.595
Afatinib	14.0					30.0		0.90	0.62-1.31	
Type of EGFR mutation										
Others	9.0	0.138				22.0	0.047	1		0.028
Exon 19 deletion	12.0					30.0		0.72	0.54-0.97	

*PFS* Progression-free survival, *OS* Median overall survival, *HR* hazard ratio, *CI* Confidence interval, *ECOG* Eastern Cooperative Oncology Group, *PS* Performance status, *TKI* Tyrosine kinase inhibitor, *EGFR* Epidermal growth factor receptor; *M* Months (median), *p p*-value

^*^Log-rank test †Cox proportional-hazards regression model

In patients with EGFR exon 19 deletion, significant differences were not observed in median PFS (12 vs. 12 months, *p*=0.868) and OS (31 vs. 28 months, *p*=0.361) between patients treated with afatinib and those treated with first-generation TKIs. However, afatinib resulted in significantly better PFS (15 vs. 9 months, *p*=0.042) and OS trend (27 vs. 19 months, *p*=0.069) compared to first-generation TKIs in patients with other EGFR mutations (Table 3). In patients receiving first-generation EGFR-TKIs, EGFR exon 19 deletion was significantly associated with better median PFS (12 vs. 9 months, *p*=0.031) and OS (28 vs. 19 months, *p*=0.045) compared to other mutations, while there was no difference in median PFS and OS based on EGFR mutation subtypes in those treated with afatinib (Table 3).

**Table 3:** Outcomes according to types of EGFR mutation and tyrosine kinase inhibitors

	**Type of EGFR mutation**	**Type of TKI**	**M**	p*	**Type of TKI**	**Type of EGFR mutation**	**M**	p*
Median PFS	Exon 19 deletion	1^st^ generation	12.0	0.868	1^st^ generation	Exon 19 deletion	12.0	0.031
		Afatinib	12.0			Others	9.0	
	Others	1^st^ generation	9.0	0.042	Afatinib	Exon 19 deletion	12.0	0.305
		Afatinib	15.0			Others	15.0	
Median OS	Exon 19 deletion	1^st^ generation	28.0	0.361	1^st^ generation	Exon 19 deletion	28.0	0.045
		Afatinib	31.0			Others	19.0	
	Others	1^st^ generation	19.0	0.069	Afatinib	Exon 19 deletion	31.0	0.604
		Afatinib	27.0			Others	27.0	

*EGFR* Epidermal growth factor receptor, *TKI* Tyrosine kinase inhibitor, *M* months (median), *p p*-value, *OS* Overall survival, *PFS* Progression-free survival

^*^Log-rank test

**Fig. 3:**
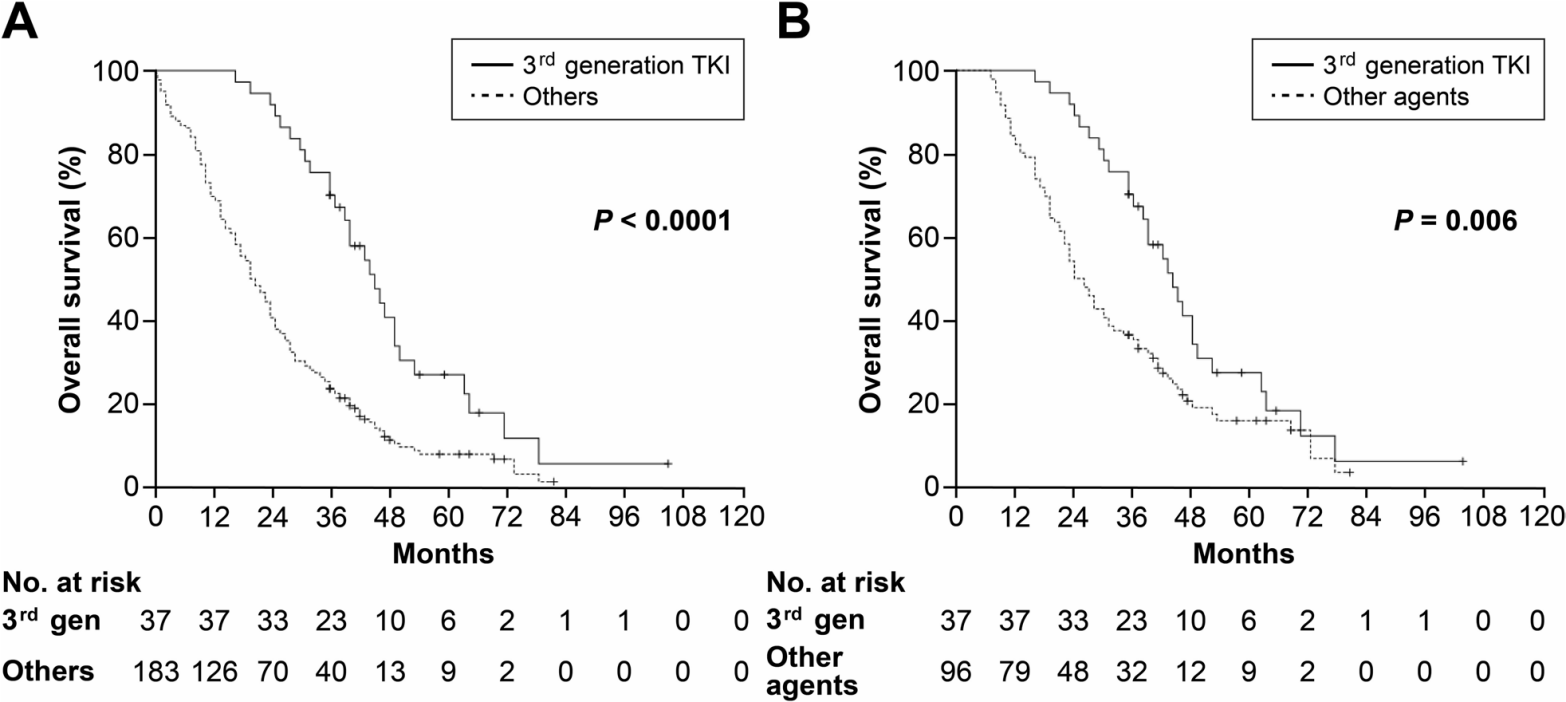
Overall survival from the start of 1st line treatment according to the 2nd or further line agents in patients with progressive disease after EGFR TKI. **A** Exon 19 deletion: 124 patients, others: 96 patients (L858R: 74, uncommon mutations: 17, dual mutations: 5) and (**B**) Exon 19 deletion: 81 patients, others: 52 patients (L858R: 39, uncommon mutations: 12, dual mutation: 1). Censoring was indicated by vertical lines

Of the patients who experienced disease progression after first-line EGFR-TKI treatment, those treated with third-generation TKIs demonstrated significantly longer median OS (44 months) from the start of first-line treatment compared to others (183 patients, 20 months, p<0.0001) as well as those who received cytotoxic agents with or without first- or second-generation TKIs (96 patients, 24 months, *p*=0.006) (Fig. 3).

## Discussion

In several retrospective/prospective studies and meta-analyses of those studies, first-generation EGFR-TKI therapy has revealed more favorable outcomes in patients with EGFR exon 19 deletion when compared to those with L858R mutation, especially in terms of PFS [21, 24, 26, 28]. Regarding second-generation EGFR-TKIs, an OS survival benefit of afatinib treatment was observed in patients with exon 19 deletion but not those with L858R mutation in a combined analysis of phase III studies (LUX-Lung 3, LUX-Lung 6) comparing cisplatin doublet chemotherapy with afatinib as a first-line setting [40]. A few molecular mechanisms were suggested, including higher drug-binding affinity [41, 42], different downstream signaling after drug treatment, and lesser baseline combined T790M mutation in exon 19 deletion [28], for the better efficacy of EGFR-TKIs in exon 19 deletion compared with L858R mutation [41–43]. However, subsequent retrospective studies and a metanalysis including afatinib and dacomitinib showed no significant differences in outcomes, especially OS, based on the type of EGFR mutation in first-line setting [27, 29, 36]. Similarly, in our cohort, patients with EGFR exon 19 deletion did not show significantly better outcomes compared with those with other mutations when treated with afatinib, while EGFR exon 19 deletion showed longer PFS and OS in patients treated with first-generation EGFR-TKIs, with an independently favorable prognostic significance of exon 19 deletion in terms of OS for all patients. Although the difference in PFS and OS between patients with EGFR exon 19 deletion and other mutations treated with first-generation TKIs is rather small compared with that reported in a previous study on patients with first-line gefitinib treatment, probably due to the longer follow-up duration, which resulted in progression in the majority of patients, the trend toward favorable clinical outcomes in patients with EGFR exon 19 deletion has been observed consistently [21]. The lack of improved PFS in patients with exon 19 deletion in the entire cohort may be explained by no significant difference in PFS based on mutation type in the afatinib group. Moreover, the significantly higher proportion of patients treated with third-generation TKIs after progression in exon 19 deletion compared with other mutations may be one of the possible explanations for favorable OS in patients with exon 19 deletion [44]. However, this result should be validated in further trials including larger numbers of patients.

Furthermore, in our study, patients with EGFR exon 19 deletion revealed almost similar median PFS and OS when they received either first-generation EGFR-TKIs or afatinib, while significantly longer median PFS and a better OS trend were observed in patients with other EGFR mutations receiving afatinib. It remains unclear whether the clinical efficacy of second-generation TKIs is superior to that of first-generation TKIs, as only dacomitinib has demonstrated an OS benefit compared to gefitinib [20]. Moreover, with second-generation TKIs, even with dose modification, the incidence of overall and grade ≥ 3 adverse events resulting in negative effects on patients’ quality of life (e.g., skin toxicity and diarrhea) is usually higher than that with first-generation TKIs [16–18, 45]. These concerns about the toxicity of afatinib may be reflected in the higher proportion of younger patients and the better performance status of patients treated with afatinib in the present study cohort. Because a proper agent must be selected based on the risk–benefit balance for each patient in clinical practice, the results of present study suggest that first-generation TKIs can be used more safely in poor performance status or elderly patients without compromising clinical efficacy compared to second-generation TKIs, especially those with exon 19 deletion.

In the FLAURA trial, osimertinib resulted in significant prolongation of PFS with a marginal OS benefit compared to first-generation EGFR-TKIs [10, 11]. However, in Asian patients and those with EGFR L858R mutation, OS benefit of osimertinib was not observed [11]. In the present study, patients treated with third-generation TKIs after first- or second-generation TKI failure showed a median OS of 44 months from the start of first-line therapy, comparable to that of osimertinib (38.6 months) in the FLAURA trial [11]. Considering that prospective data directly comparing second- and third-generation EGFR-TKIs are not currently available, the results of a few studies including ours suggest that first-line second-generation TKIs and sequential third-generation EGFR-TKI treatment may be an effective therapeutic strategy, especially in patients with EGFR L868R mutation [20, 46]. Overall, first-line first- or second-generation EGFR-TKIs may still be a reasonable choice in routine practice due to its cost-effectiveness [47] in countries where first-line osimertinib is not reimbursable, such as Korea.

The current study demonstrated that EGFR exon 19 deletion was associated independently with favorable OS in advanced NSCLC patients treated with first-line EGFR-TKIs. To the best of our knowledge, current study is the first one showing a significantly favorable OS in patients with EGFR exon 19 deletion, when compared with other mutations in advanced NSCLC patients treated with either first- or second-generation EGFR-TKIs as first-line therapy. Moreover, as this study analyzed every EGFR mutation-positive patient who received first-line first- or second-generation EGFR-TKIs therapy during the defined period with a fairly long follow-up duration (minimum follow-up duration of survivors: 35 months), it reflected the patient outcomes of everyday clinical practice.

However, several limitations were included in this study. First, it was retrospective and performed at a single institution. Second, the number of patients who received third-generation TKIs as second- or further-line therapy was small, as second- or further-line osimertinib treatment has been reimbursable by the Korean national health insurance system since late 2017. Finally, the collection of treatment-related adverse events was not planned considering the retrospective nature of this study.

Nonetheless, several clinical implications can be suggested by the results of our study. First, first-generation EGFR-TKIs could still be recommended as a first-line palliative treatment for NSCLC with EGFR exon 19 deletion, especially in elderly and fragile patients. Second, patients and their families could receive more precise explanations regarding the outcomes and further treatment options after EGFR-TKI therapy based on the types of EGFR mutation. Finally, this study recommends that further clinical trials with EGFR-TKIs should still consider the types of EGFR mutation as a stratification factor.

## Conclusions

The EGFR exon 19 deletion was correlated with favorable OS in advanced NSCLC treated with first-line EGFR-TKIs. Moreover, in patients with exon 19 deletion, first-generation TKIs seem to be a reasonable treatment option if osimertinib is unavailable.

## Data Availability

The datasets generated and/or analyzed during the current study are not publicly available due to the confidentiality of the data of patient but are available from the corresponding author on reasonable request.

## Abbreviations

NSCLC: Non-small cell lung cancer
OS: Overall survival
EGFR: Epidermal growth factor receptor
TKIs: Tyrosine kinase inhibitors
PFS: Progression-free survival
AJCC: American Joint Committee on Cancer
PS: Performance status
ECOG: Eastern Cooperative Oncology Group
FFPE: Formalin-fixed, paraffin embedded
PCR: Polymerase chain reaction
PNA-LNA: Peptide nucleic acid-locked nucleic acid
IRB: Institutional review board
